# Blastocyst hatching site is regularly distributed and does not influence foetal development in mice

**DOI:** 10.1038/s41598-020-59424-2

**Published:** 2020-02-12

**Authors:** Shu-Jun Liu, Jia-Bo Sun, Xin Hao, Zhe Han, Xin Wen, Xing-Yue Wang, Cheng-Jie Zhou, Cheng-Guang Liang

**Affiliations:** State Key Laboratory of Reproductive Regulation & Breeding of Grassland Livestock, School of Life Sciences, Inner Mongolia University, Hohhot, Inner Mongolia People’s Republic of China

**Keywords:** Biological techniques, Developmental biology

## Abstract

Hatching out from the zona pellucida (ZP) is a crucial step for blastocyst implantation and development. However, it is still unknown whether the location of the hatching site relative to the inner cell mass (ICM) affects embryo implantation and foetal development. Here, we classified hatching blastocysts into three categories, 0° ≤ θ ≤ 30°, 30° < θ ≤ 60°, and 60° < θ ≤ 90°, in which θ is determined based on the relative position of the hatching site to the arc midpoint of the ICM. Non-surgical embryo transfer (NSET) devices were employed to evaluate blastocyst implantation and embryo development. Of 1,827 hatching blastocysts, 43.84%, 30.60%, and 21.67% were categorized as 30° < θ ≤ 60°, 0° ≤ θ ≤ 30°, and 60° < θ ≤ 90°, respectively. Embryos with different hatching sites showed no distinct differences in blastocyst implantation; surrogate female pregnancy; embryo development to term; litter size, or offspring survival, gender, or body weight. Our results indicate that mouse blastocyst hatching site is not randomly distributed. Embryo implantation and development are not correlated with the blastocyst hatching site in mice. Thus, assessment of the blastocyst hatching site should not be recommended to evaluate mouse blastocyst implantation and developmental potential.

## Introduction

Hatching is a prerequisite for mammalian blastocyst implantation, whereby the embryo at the blastocyst stage squeezes out from its zona pellucida (ZP)^1^. Impairment of blastocyst hatching leads to implantation failure or early pregnancy loss^2–4^. Embryo implantation, which follows blastocyst hatching, establishes the pregnancy. Successful embryo implantation is determined by three factors: embryo quality, the receptivity of the uterine endometrium, and the intricate relationship between the two^5,6^.

It is still a subject of debate whether the blastocyst hatching site has an influence on the final hatched rate. Observational studies demonstrated that the natural hatching site in humans usually develops at a position close to the blastocyst inner cell mass (ICM)^7,8^. In mice, the site of zona opening by laser did not affect the complete hatching or the number of cells in completely hatched blastocysts^9^. However, artificial opening at a site in close proximity to the ICM resulted in a higher rate of complete hatching in human blastocysts^8^. Embryos generated by *in vitro* fertilization (IVF) or intracytoplasmic sperm injection (ICSI) have two distinct embryo hatching patterns, including small trophectoderm projections penetrating the ZP and regular rupture of the ZP followed by extrusion of the blastocyst^10^. Although these two hatching patterns were both observed in IVF and ICSI embryos, they have no effect on embryo implantation. In human vitrified-warmed blastocysts, the assisted hatching (AH) site (near or opposite to the ICM) did not affect blastocyst implantation or subsequent pregnancy and live birth rates^11^. Conversely, others found that ZP opening close to the ICM was associated with a higher implantation and pregnancy rate in humans^12^. Therefore, it is still not known if the hatching site determines embryo implantation and foetal development.

Although the observation of *in vitro* AH has been recorded, the relationship between the natural hatching site and blastocyst implantation has not been investigated. Moreover, no studies concerning the relationship between the natural blastocyst hatching site and further foetal developmental potential were conducted. The purpose of this study is to assess the implantation and foetal development of blastocysts with different hatching sites after transfer by a non-surgical approach. The methodology involved in our study and the classification of blastocyst hatching sites may provide guidance for future human blastocyst hatching analysis.

## Results

### Sorting and hatching rate of blastocysts

**Figure 1:**
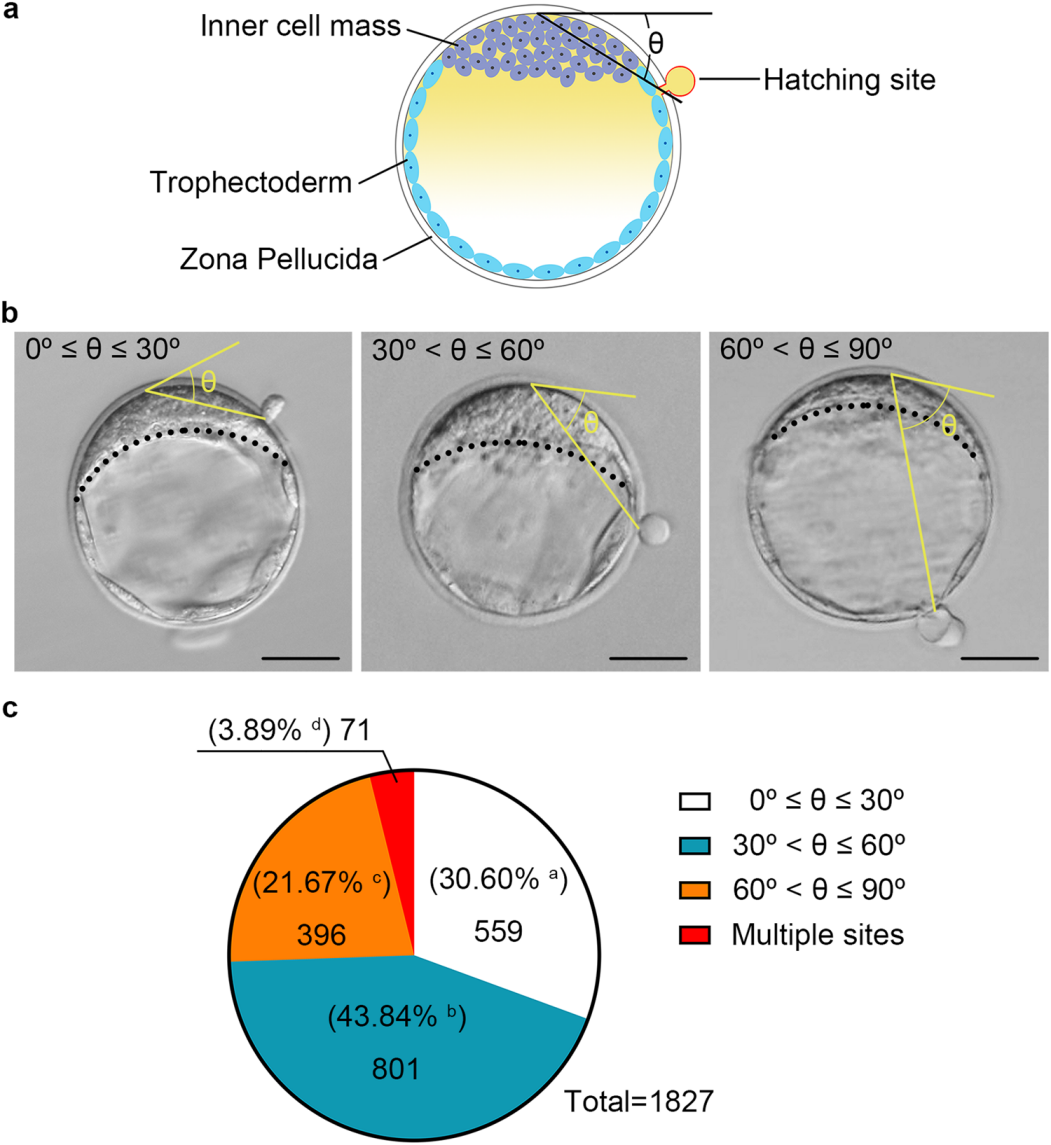
Effect of hatching site on the hatching process. (**a**) Schematic of the morphology of angle measurements of blastocyst hatching sites. During the measurement, two lines were drawn, the tangent line of the ICM arc midpoint and a straight line connecting the hatching site to the ICM arc midpoint. The acute angle between two straight lines is the hatching angle θ. (**b**) Sorting of blastocysts. Left, middle and right are representative of the hatching blastocysts 0° ≤ θ ≤ 30°, 30° < θ ≤ 60°, and 60° < θ ≤ 90°, respectively. The dotted line marks the ICM circle arc. Scale bar = 50 μm. (**c**) Percentage of hatching blastocysts 100 hours after administration of human chorionic gonadotropin (hCG) to the 1,827 blastocysts with natural hatching sites. Data were analysed by chi-square test. Percentages without a common letter (**a**–**d**) differs (*P* < 10^−4^).

Among the 1,827 hatching blastocysts, 30.60% (559/1,827) were classified as 0° ≤ θ ≤ 30°; 43.84% (801/1,827) were classified as 30° < θ ≤ 60°; and 21.67% (396/1,827) were classified as 60° < θ ≤ 90°. In addition, 3.89% (71/1,827) were classified as having multiple hatching sites (Fig. 1c). Statistically significant differences existed among the four classifications (*P* < 10^−4^). The results showed that in CD1 mice the percentage of blastocysts hatched from the site 30° < θ ≤ 60° was significantly higher than that from other sites.

### Hatching sites do not affect blastocyst implantation and development in utero

To assess the effect of blastocyst hatching site on the implantation rate and embryo developmental potential *in utero*, we transferred the hatching blastocysts to pseudopregnant recipients using the NSET devices. No differences were observed in the percentage of pseudopregnant recipients with implanted blastocysts (Table 1) or in the percentage of implanted blastocysts among the three groups (Table 2). There were no statistically significant differences in the percentage of pregnant surrogate females among the three groups (Table 3). Moreover, the percentage of blastocysts developed to term was also comparable among the three groups (Table 4).

**Table 1:** Pseudopregnant recipients implanted with blastocysts.

Angle	0° ≤ θ ≤ 30°	30° < θ ≤ 60°	60° < θ ≤ 90°
Number of pseudopregnant females subjected to blastocyst transfer	16	20	13
Number of pseudopregnant females with implanted blastocysts	13	14	10
% Implantation of pseudopregnant females	81.25^a^	70.00^a^	76.92^a^

Data were analysed by chi-square test. ^a^Values within a line denote no significant difference among groups (P > 0.05).

**Table 2:** Implanted blastocysts.

Angle	0° ≤ θ ≤ 30°	30° < θ ≤ 60°	60° < θ ≤ 90°
Number of total transferred blastocysts	250	350	195
Number of implanted blastocysts	203	273	144
% Implantation blastocysts	81.20^a^	78.00^a^	73.85^a^

Data were analysed by chi-square test. ^a^Values within a line denote no significant difference among groups (P > 0.05).

**Table 3:** Pregnant surrogate females 14 days after blastocyst transplantation.

Angle	0° ≤ θ ≤ 30°	30° < θ ≤ 60°	60° < θ ≤ 90°
Number of surrogate females for blastocyst transfer	17	20	13
Number of surrogate females pregnant for 14 days	12	13	8
% Pregnant surrogate females 14 days after transplantation	70.59^a^	65.00^a^	61.54^a^

Data were analysed by chi-square test. ^a^Values within a line denote no significant difference among groups (P > 0.05).

**Table 4:** Blastocysts developed to term.

Angle	0° ≤ θ ≤ 30°	30° < θ ≤ 60°	60° < θ ≤ 90°
Number of transplanted blastocysts	255	300	195
Number of delivered pups	84	92	61
% Blastocysts developed to term	32.94^a^	30.67^a^	31.28^a^

Data were analysed by chi-square test. ^a^Values within a line denote no significant difference among groups (P > 0.05).

### Hatching site does not influence foetal birth and offspring growth

**Figure 2:**
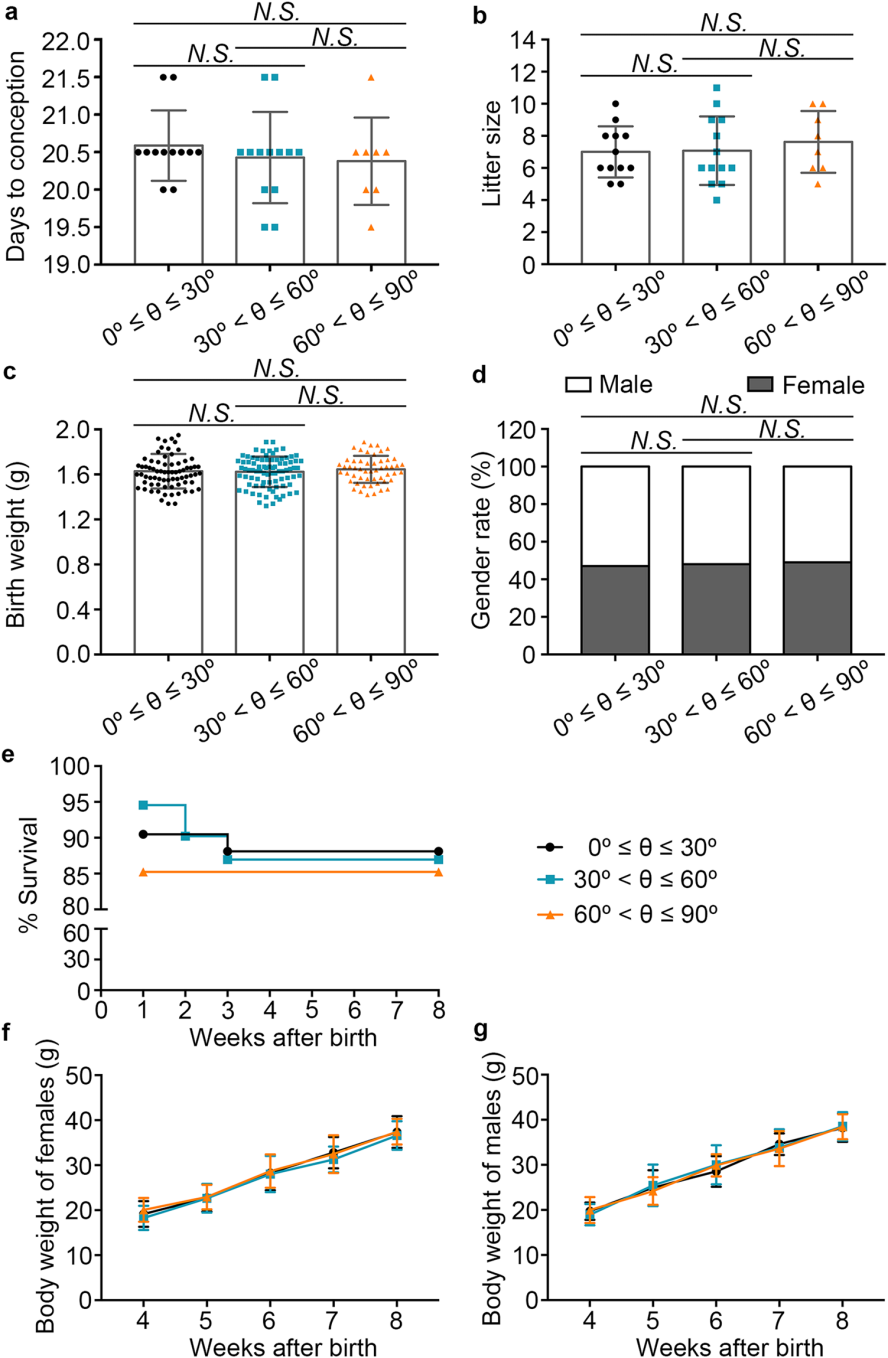
Hatching site did not affect foetal outgrowth and survival rate of the offspring. (**a**) Days to conception of the pseudopregnant females. (**b**) Litter size. (**c**) Offspring birth weight. (**d**) Offspring gender ratio. (**e**) Offspring survival rate. (**f**) Body weight of female offspring from age of 4 to 8 weeks. (**g**) Body weight of male offspring from age of 4 to 8 weeks. For (**a**,**b**), at least eight pregnant females were included. For **c**, at least 53 newborn pups were counted. For (**a**–**c**,**f**,**g**), the data are presented as mean ± standard deviation (SD) and differences between groups were evaluated with Tukey’s multiple comparison tests. For (**d**), the data were evaluated using a chi-square test. For (**e**) differences between each group were evaluated with log-rank (Mantel-Cox) test. *N.S*., not significant.

To further understand the effect of hatching site on fetal growth, days to conception was recorded, and the results showed that there were no significant changes for the pregnancy days among the groups (Fig. 2a). Litter size (Fig. 2b), birth weight (Fig. 2c), and the gender ratio (Fig. 2d) were also comparable among the three groups. In terms of the offspring survival rate after delivery, 15% of pups died in the first week in the group of 60° < θ ≤ 90°, which is higher than that for 0° ≤ θ ≤ 30° (10%) and 30° < θ ≤ 60° (5%). However, there is no statistically significant difference among the three groups (Fig. 2e, Table 5). After weaning, male and female offspring were separated according to sex. The body weight of pups from 4 weeks to 8 weeks of age was recorded. The results showed that there was no significant difference in terms of body weight increase in females or males among the three groups (Fig. 2f,g, Tables 6, 7), suggesting blastocyst hatching site had no impact on offspring growth.

**Table 5:** Survival rates of offspring.

Weeks after birth	Angle		
	0° ≤ θ ≤ 30°	30° < θ ≤ 60°	60° < θ ≤ 90°
1	90.48%^a^	94.57%^a^	85.25%^a^
2	90.48%^a^	90.22%^a^	85.25%^a^
3	88.10%^a^	86.96%^a^	85.25%^a^
4	88.10%^a^	86.96%^a^	85.25%^a^
5	88.10%^a^	86.96%^a^	85.25%^a^
6	88.10%^a^	86.96%^a^	85.25%^a^
7	88.10%^a^	86.96%^a^	85.25%^a^
8	88.10%^a^	86.96%^a^	85.25%^a^

Data were analysed by log-rank (Mantel-Cox) test. ^a^Values within a line denote no significant difference among groups (P > 0.05).

**Table 6:** Body weight of female offspring.

Weeks after birth	Angle		
	0° ≤ θ ≤ 30° (g)	30° < θ ≤ 60° (g)	60° < θ ≤ 90° (g)
4	19.16 ± 2.85^a^	18.29 ± 2.69^a^	20.08 ± 2.63^a^
5	22.76 ± 3.02^a^	22.66 ± 3.20^a^	22.93 ± 2.73^a^
6	28.25 ± 3.83^a^	29.03 ± 4.02^a^	28.70 ± 3.72^a^
7	32.79 ± 3.47^a^	31.28 ± 2.88^a^	32.47 ± 4.19^a^
8	37.37 ± 3.53^a^	36.60 ± 3.18^a^	37.45 ± 2.85^a^

Data are presented as mean ± SD and were processed by Tukey multiple comparisons test. ^a^Values within a line denote no significant difference among groups (P > 0.05).

**Table 7:** Body weight of male offspring.

Weeks after birth	Angle		
	0° ≤ θ ≤ 30° (g)	30° < θ ≤ 60° (g)	60° < θ ≤ 90° (g)
4	19.73 ± 1.91^a^	18.96 ± 2.37^a^	19.99 ± 2.87^a^
5	24.94 ± 3.88^a^	25.45 ± 4.62^a^	24.20 ± 3.06^a^
6	28.55 ± 3.38^a^	30.02 ± 4.32^a^	29.90 ± 2.47^a^
7	34.59 ± 2.40^a^	33.81 ± 4.10^a^	33.59 ± 3.87^a^
8	38.23 ± 3.14^a^	38.57 ± 3.14^a^	38.44 ± 2.77^a^

Data are presented as mean ± SD and were processed by Tukey multiple comparisons test. ^a^Values within a line denote no significant difference among groups (P > 0.05).

## Discussion

We assessed the effect of blastocyst hatching site on embryo implantation and foetal development in mice. The results showed that significantly more blastocysts hatched from the site 30° < θ ≤ 60°, whereas different hatching sites had no influence on subsequent implantation; surrogate female pregnancy; embryonic development to term; litter size; or offspring survival, gender, or body weight.

In humans, a previous study showed that fresh blastocysts usually hatch from the site opposite the ICM, although a few choose to hatch near the ICM or elsewhere^13^; however, the correlation between hatching site and birth outcome was not evaluated. Conversely, another study employed fresh bovine embryos and showed that there was nearly an equal probability that hatching would occur ipsilateral or contralateral to the ICM^14^. Combined with our results on blastocyst hatching site inclination, we presume that different species have variable preferences in terms of blastocyst hatching site. Our results illustrate that the preference for blastocyst hatching site is not correlated with subsequent embryonic development in mice. However, due to the limitations and ethical restrictions on human blastocyst usage, our experimental design cannot be conducted with human material, and our conclusion may not be applicable to humans. Whether the same situation exists in humans and other species needs further investigation. It is worth mentioning that the methodology involved in our study and the classification of the blastocyst hatching site may provide guidance for future human blastocyst hatching analysis.

In clinical research, AH in pre-implantation embryos is often used to facilitate blastocyst hatching and embryo implantation. Mechanical methods, chemical drug treatment, and laser or piezo penetration are widely used as AH approaches. Penetrating the zona or making the zona thinner can be performed with these methods. Location selection on the zona during AH has been studied and different labs have generated controversial results. Some researchers performing AH at a site close to the ICM observed a higher rate of complete hatching in human vitrified blastocysts^8^. Interestingly, further study found the AH site had no influence on implantation, clinical pregnancy, or live birth rate^11^. Others found the site of AH did not influence the rate of completed hatching by blastocysts in mice^9^. Consistent with these findings, our results show that hatching site does not influence the hatching rate, implantation, pregnancy, or live birth rate in mice. There is also the possibility that blastocysts of different species or generated by different approaches, *i.e*., fresh or vitrified, may have different hatching sites.

Considering the contribution of the uterus during blastocyst hatching^15,16^, it would be ideal to monitor the hatching progress in an *in vivo* culture model. However, there is no suitable method to determine the blastocyst hatching site *in vivo*. Thus, it is necessary to flush out the blastocysts from the horn of the uterus and culture them *in vitro* for a short time in order to measure the angle between the hatching site and the ICM arc midpoint. In our protocol, we tried our best to minimize the duration of *in vitro* culture and transferred blastocysts to the pseudopregnant recipient immediately after the hatching site was determined. Nevertheless, even short time culture *in vitro* cannot represent the hatching process *in vivo*. One thing that needs to be mentioned is that, clinically, blastocysts generated by *in vitro* culture often undergo embryo quality evaluation before transfer to the uterus. The experimental design and the methodology in our study may apply to human infertility investigation. We believe that the combination of our data in mice and the broad observational data from IVF labs around the world will provide more applicable guidance for human-assisted reproductive technology.

In order to minimize the disadvantages caused by surgical embryo transfer, such as invasive surgery and side effects of anaesthesia, we used the NSET device, which was invented in 2009, as an alternative^17,18^. The live birth rate with NSET in a previous study is in the range of 10.0% to 35.6%^19^. In other studies, it was reported that the live birth rate is 33.3% after NSET^20^. In our study, the percentage of blastocysts developed to term was around 30%, which is comparable to the previous studies. Interestingly, whether the NSET is a suitable approach for all mouse strains and whether it benefits blastocyst implantation and foetal development still needs further investigation.

## Conclusion

We found that blastocysts from the site 30° < θ ≤ 60° had a significantly higher proportion of hatching in mice. However, the hatching site had no influence on implantation and foetal potential development. Thus, the assessment of blastocyst hatching site should not be recommended for evaluating mouse implantation and embryo developmental potential.

## Methods

### Chemicals, instruments, and reagents

Reagents and anaesthetics were from Sigma-Aldrich (St. Louis, MO, USA) except where otherwise noted. The materials used in this study included pregnant mare serum gonadotropin (PMSG) and human chorionic gonadotropin (hCG) (both Sansheng, NingBo, China); petri dishes (BD Falcon, Franklin Lakes, NJ, USA); thin glass tubes (Zhengtianyi, Beijing, China); stereomicroscope (Nikon, Tokyo, Japan); CO_2_ incubator (Sanyo, Osaka, Japan); non-surgical embryo transfer (NSET) device (ParaTechs, Lexington, KY, USA).

### Ethics statement

All animal experimental protocols were according to the guide for the National Research Council Guide for the Care and Use of Laboratory Animals and were approved by the Institutional Animal Care and Use Committee at Inner Mongolia University (Approval number: SYXK 2014-0002).

### Animal treatments

CD1 female mice 8 weeks of age and male mice 10 weeks of age were purchased from the Animal Research Centre at Inner Mongolia University and housed in cages at 23 °C with a photoperiod of 12 h light and 12 h darkness with free access to food and water.

Male mice with good mating records were used for vasectomy. Male mice were anesthetized by intraperitoneal injection with ketamine (0.1 mg/g: 0.01 ml/g of a 10 mg/ml solution) and xylazine (0.01 mg/g: 0.005 ml/g of a 2 mg/ml solution) followed by vasectomy surgery according to Bermejo-Alvarez^21^. After 2 weeks of recovery, male mice that had been proved to be infertile were used for mating to prepare pseudopregnant recipients.

The pseudopregnant recipient females used for embryo transfer were obtained by natural mating with vasectomized males. Female mice were checked each morning for vaginal copulation plugs. Plug-positive females were chosen and recorded as 0.5 day post coitum (dpc) pseudopregnant recipients. Pseudopregnant recipients at 2.5 dpc were used for blastocyst transplantation.

For blastocyst collection, female mice were injected with 7 IU of PMSG followed 48 hours later by 7 IU of hCG. Superovulated females were mated with fertile males. The following morning, females were examined for the presence of vaginal copulation plugs and considered to be 0.5 dpc.

### Blastocyst collection and culture

To obtain blastocysts developed *in vivo*, the donors at 3.5 dpc were sacrificed by cervical dislocation. Blastocysts were flushed from the horn of the uterus with M2 medium containing 4% bovine serum albumin (BSA) and cultured in Chatot, Ziomek, Bavister (CZB) medium supplemented with 3% BSA in a humidified atmosphere of 5% CO_2_ at 37 °C.

### Definition and measurement of blastocyst hatching angle

Hatching status of blastocysts was recorded at 94 hours, 96 hours, 98 hours, and 100 hours after hCG injection. A sketch demonstrating measurement of the hatching site angle is shown in Fig. 1a. Blastocysts were rotated with a Pasteur pipet to focus both ICM and the hatching site. The acute angle formed by the line across the ICM centre point and the hatching site and the tangential line across the ICM arc midpoint was recorded as the hatching angle (θ). According to the hatching angle (0° ≤ θ ≤ 30°, 30° < θ ≤ 60°, and 60° < θ ≤ 90°), blastocysts were divided into three categories. Representative blastocysts with different hatching angles are shown in Fig. 1b.

### Non-surgical embryo transfer

Blastocysts transfer with the NSET device was performed as described^17,19^. Briefly, the NSET device was loaded with 16 blastocysts for each recipient and the tip was inserted to the edge of the speculum. The blastocysts were released into the uterus by pushing the plunger all the way down.

### Observation of implantation site

At 12.5 dpc, the recipient mice were anesthetized with ketamine and xylazine followed by tail vein injection with 0.2 ml Trypan blue (1% in normal saline). Three minutes after the injection, mice were sacrificed and the blastocyst implantation sites marked with Trypan blue were counted under a stereomicroscope.

### Pregnancy and offspring assessment

At 16.5 dpc, the recipient mice were checked and those with abdominal prominence were determined to be pregnant. Litter size and birth weights of pups were recorded immediately after delivery. Body weight was measured and recorded once a week. The offspring were weaned at 3 weeks of age after gender distinction.

### Statistical analysis

Chi-square tests in Microsoft Excel software (Microsoft Corporation, Washington, USA) were used to analyse the percentage of hatching blastocysts and gender ratio. Data for days to conception, litter size, offspring birth weight and body weight are presented as mean ± standard deviation (SD), and differences between each group were evaluated using One-way ANOVA with Tukey’s multiple post-hoc comparison test in GraphPad Prism 7.0 (GraphPad Software Inc., San Diego, CA, USA). The differences in survival rate were evaluated with the log-rank (Mantel-Cox) test in GraphPad Prism 7.0. *P* < 0.05 was considered statistically significant.

## Data Availability

The datasets generated and/or analysed during the current study are available from the corresponding author on reasonable request.
